# A Case Requiring Differential Diagnosis of Synovitis, Acne, Pustulosis, Hyperostosis, Osteomyelitis Syndrome in the Evaluation of Bone Lesions in Early Breast Cancer

**DOI:** 10.70352/scrj.cr.25-0822

**Published:** 2026-04-24

**Authors:** Mayuka Mori, Masayuki Kikuchi, Rika Miyabe, Hidenori Kita, Junko Kobayashi, Koji Atsuta, Tsunehiro Shintani

**Affiliations:** 1 Department of Surgery, Japanese Red Cross Shizuoka Hospital, Shizuoka, Shizuoka, Japan; 2 Tosen Clinic, Shizuoka, Shizuoka, Japan

**Keywords:** SAPHO syndrome, breast cancer, bone metastasis

## Abstract

**INTRODUCTION:**

Synovitis, Acne, Pustulosis, Hyperostosis, Osteomyelitis (SAPHO) syndrome is a rare disorder characterized by bone lesions that primarily affect the anterior thoracic wall. These lesions can be difficult to differentiate from bone metastases of malignant tumors. Reports of SAPHO syndrome complicating breast cancer are scarce, necessitating careful diagnostic consideration.

**CASE PRESENTATION:**

An 81-year-old woman with a past history of palmoplantar pustulosis presented with of a mass in her right breast. After a biopsy and diagnosis of mucinous carcinoma of the right breast, CT and bone scintigraphy were performed to check for distant metastases. This screening revealed multiple sclerotic bone lesions and abnormal radiotracer uptake, including a bull’s head sign at the sternoclavicular joint, a feature distinguishing it from breast cancer bone metastases. Considering her history of skin lesions, clinical course, and characteristic imaging findings, a multidisciplinary team concluded that these bone lesions were not distant metastases but due to SAPHO syndrome. After partial mastectomy of the right breast and sentinel lymph node biopsy, the patient underwent adjuvant therapy with no recurrence at 16 months post-procedure.

**CONCLUSIONS:**

In early-stage breast cancer, particularly mucinous carcinoma, which has a low metastatic risk, multiple bone lesions should not be deemed bone metastases based on imaging findings alone. Integrating clinical information, including prior anterior thoracic lesions and skin symptoms, can reduce invasive testing and unnecessary treatment. This case demonstrates the importance of considering SAPHO syndrome as a key differential diagnosis in the management of breast cancer.

## Abbreviations

CA
: cancer antigen
CEA
: carcinoembryonic antigen
ER
: estrogen receptor
PgR
: progesterone receptor
SAPHO
: Synovitis, Acne, Pustulosis, Hyperostosis, Osteomyelitis

## INTRODUCTION

SAPHO syndrome is a rare disorder characterized by sclerotic bone lesions in the anterior thoracic region and skin lesions such as palmoplantar pustulosis. SAPHO syndrome presents with bone sclerosis and thickening visible by CT, bone marrow edema detectable by MRI, and high uptake of radiotracer upon bone scintigraphy. Because the imaging resembles that of breast cancer bone metastases, differential diagnosis is notoriously difficult in patients with malignancies.

The presence or absence of bone metastases in breast cancer significantly influences staging and treatment decisions. However, misdiagnosing bone lesions caused by SAPHO syndrome as metastases risks unnecessary chemo- or endocrine therapy. Reports of breast cancer patients with concomitant SAPHO syndrome are extremely rare, and consequently, there is limited clinical guidance for differential diagnosis.

Here, we report a case of early-stage breast cancer in which multiple sclerotic bone lesions were identified by CT and bone scintigraphy, making the differentiation of SAPHO syndrome from breast cancer bone metastasis challenging. However, based on the patient’s history and imaging characteristics, SAPHO syndrome was ultimately diagnosed.

## CASE PRESENTATION

An 81-year-old woman with a history of palmoplantar pustulosis and pain around the sternoclavicular region presented at our breast surgery outpatient clinic with her main complaint: a right breast mass.

**Fig. 1:**
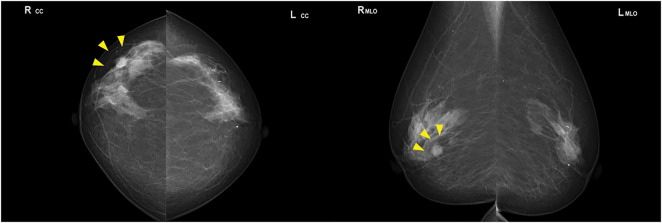
Mammography image. The wedge markers revealed a finely serrated mass in the right upper quadrant, which was classified as category 4. cc, cranio-caudal; MLO, medio-lateral oblique view

**Fig. 2:**
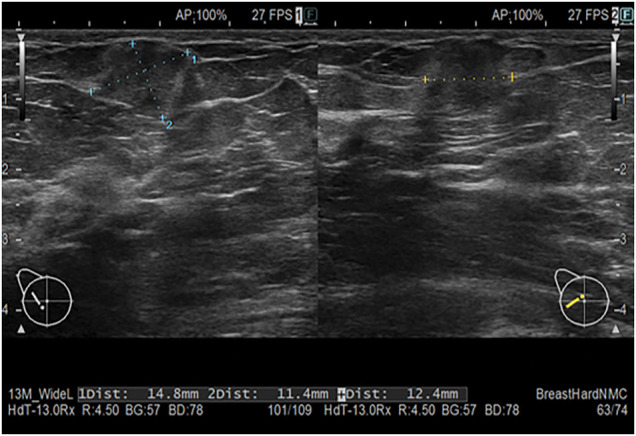
US image. A 14.8 × 12.4 × 11.4-mm solid mass was found at the 9 o’clock position of the right breast, which was characterized by mildly heterogeneous internal echogenicity, ill-defined borders, and irregular margins. The lesion showed a discontinuous anterior halo, findings suggestive of breast cancer.

Mammography revealed a finely serrated mass in the right upper quadrant, which was classified as category 4 (**Fig. 1**). US revealed an approximately 1 cm^3^ solid lesion at the 9 o’clock position in the right breast that exhibited a mildly heterogeneous internal appearance, ill-defined borders, and irregular margins. The mass was haloed with a discontinuous anterior border, findings consistent with breast cancer (**Fig. 2**).

**Fig. 3:**
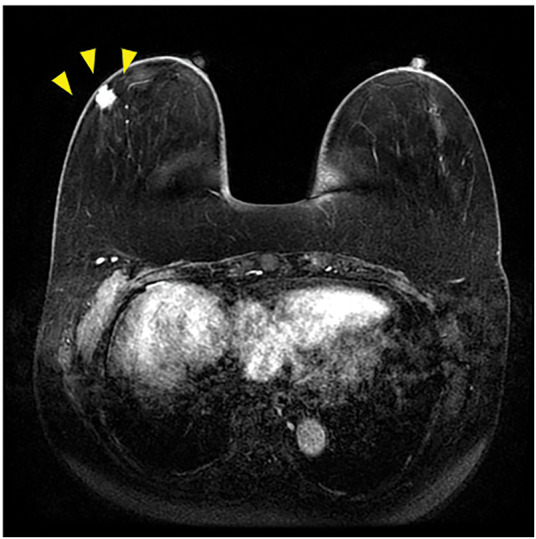
Gadolinium-enhanced MRI image. A 1.2-cm spiculated mass in contact with the skin was found in the D region of the right breast. The wedge markers indicate the lesion.

**Fig. 4:**
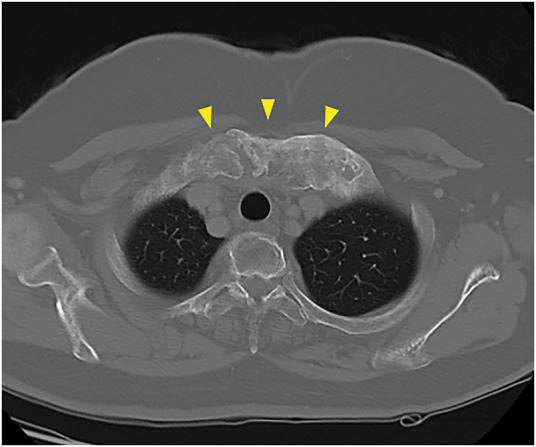
Breast CT image. A tumor with poor contrast enhancement, consistent with a primary lesion, was identified immediately beneath the skin in the C region, with no evidence of metastatic disease. Bilateral sclerosis and hyperplasia of the medial first ribs and sternum were also observed. The wedge markers indicate the lesion.

Based on the imaging results, breast cancer was suspected, and a vacuum-assisted breast biopsy was performed for pathological examination. The definitive diagnosis was mucinous carcinoma. Further screening was conducted, including blood tests, MRI to assess ductal extension, CT to assess distant metastases, and bone scintigraphy to detect any lesions. The blood tests indicated normal complete blood counts and biochemistry: CEA was 0.92 ng/mL and CA 15-3 was 8.0 U/mL, both within normal ranges. Gadolinium-enhanced MRI revealed a 1.2 cm tumor with spiculation that was in contact with the skin in the D region of the right breast. No intraductal extension was detected (**Fig. 3**). Contrast-enhanced CT indicated no lymph node, lung, or liver metastases. A poorly enhanced tumor was present immediately beneath the skin in the heterogeneously dense C region, consistent with the primary lesion. Bilateral sclerosis and hyperplasia of the medial first rib and sternum were noted (**Fig. 4**).

**Fig. 5:**
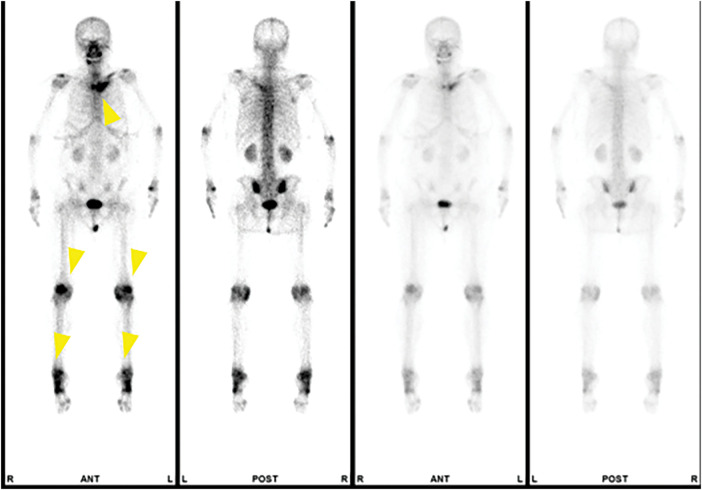
Bone scintigraphy image. The bull’s head sign was observed at the sternoclavicular joint. Additionally, abnormal accumulations consistent with bone enlargement and sclerosis on CT were found at the sternum to the distal end of the left clavicle, the spine, both knee joints, and both ankle joints. The wedge markers indicate the lesion.

Bone scintigraphy revealed a bull’s head sign at the sternoclavicular joint. Additionally, abnormal radiotracer uptake was observed from the sternum to the distal left clavicle, in the spine, and in both knee and ankle joints, consistent with the bone enlargement and sclerosis visualized by CT (**Fig. 5**). PET-CT was not performed, as it is not routinely indicated for early-stage breast cancer at our institution. CT and bone scintigraphy revealed multiple bone lesions, raising the suspicion that these were metastases from breast cancer. However, considering the patient’s history of palmoplantar pustulosis and the absence of subjective symptoms, consultations with specialists from Radiology, Orthopedics, and Dermatology were conducted. The clinical diagnosis was SAPHO syndrome, and the bone lesions were attributed to this condition. The final diagnosis was cT1bN0M0 (AJCC, 8th edition), Stage I cancer. Partial mastectomy of the right breast and a sentinel lymph node biopsy were performed. Rapid pathological analysis showed that the sentinel lymph node was negative for cancerous cells; therefore, axillary lymph node dissection was not performed. The pathological diagnosis was mucinous carcinoma, ER 100%, PgR 100%, HER2 0, Ki67 labeling index 12%, pT2 28 × 22 × 10 mm, lymphatic invasion negative (D2-40), venous invasion positive (Factor-VIII-M), HG1 (tubule formation:1, nuclear atypia:2, mitotic counts:1 (1/10 high-power field)), and pT2N0M0 (AJCC, 8th edition), pStage IIA. The right breast was preserved, and the patient underwent breast-conserving radiotherapy as an adjuvant treatment. She was also started on oral letrozole and carefully monitored as an outpatient; as of 16 months, she remained recurrence-free with no new symptoms related to SAPHO syndrome.

## DISCUSSION

First reported by Chamot et al. in 1987, SAPHO syndrome is a disease characterized by synovitis, acne vulgaris, pustulosis, hyperostosis, and osteomyelitis.^1)^ The etiology is unclear, with theories suggesting an infection by *Propionibacterium acnes* or an association with seronegative spondyloarthropathy.^2)^ Although the estimated prevalence is extremely low, at 1 in 10000 Caucasians and 0.00144 per 100000 people in Japan, the exact prevalence of SAPHO syndrome remains unknown.^3)^ The diagnosis is based mainly on the clinical signs, indicated by the presence of at least one of the inclusion criteria and none of the exclusion criteria, as described by Benhamou et al.^4)^

No standard treatment protocols are available, and current treatment options are not evidenced-based because of the rarity of the syndrome. Therapy is empirical and aimed at easing pain and modifying the inflammatory process. It includes nonsteroidal anti-inflammatory drugs as the first-line agents. Antibiotics, corticosteroids, disease-modifying antirheumatic drugs, biologics targeting tumor necrosis factor alpha or interleukin-1, and bisphosphonates have all been used with variable success.^5)^

Bone lesions in the anterior thoracic region, such as the sternoclavicular and manubrium joints, and the costosternal and costochondral junctions, are characteristic of SAPHO syndrome. Skin symptoms occur in approximately 50% of cases and often precede bone lesions by several years.^2)^

Although SAPHO syndrome is rare in patients with breast cancer, differential diagnosis is crucial because the bone lesions closely resemble bone metastases on imaging,^6)^ namely: (1) bone sclerosis and thickening on CT, (2) high T2 signal intensity on MRI, and (3) high radiotracer uptake on bone scintigraphy.^7)^ Furthermore, while the “bull’s head sign”, that is, symmetrical tracer uptake at the sternoclavicular joints and manubrium on bone scintigraphy, is characteristic of SAPHO syndrome,^2)^ it is not always present.^8)^ When multiple uptake areas are present only outside the anterior thoracic wall, differentiation from neoplastic lesions becomes difficult. The imaging features of SAPHO syndrome and breast cancer bone metastases have been described separately in previous reports. Based on these published findings, the similarities and distinguishing characteristics across imaging modalities are summarized in **Table 1** to facilitate differential diagnosis.^6,9,10)^

**Table 1:** Similarities and differences between SAPHO syndrome and breast cancer bone metastases

Category	Similarities	Differences
Clinical presentation	Bone pain may be present	SAPHO: History of palmoplantar pustulosis, acne, anterior chest wall pain
		Breast cancer metastasis: No inflammatory skin lesions
CT	Sclerotic or mixed lesions may be present	SAPHO: Osteosclerosis, cortical thickening, hyperostosis; often symmetrical anterior chest wall involvement
		Breast cancer metastasis: Frequently osteolytic or mixed lesions with cortical destruction; asymmetrical distribution
Bone scintigraphy	Increased uptake in multiple skeletal sites	SAPHO: Characteristic “bull’s head sign” in sternoclavicular region
		Breast cancer metastasis: Random focal uptake without a specific pattern
FDG PET-CT	FDG uptake in affected bones	SAPHO: Mild-to-moderate uptake; inflammatory pattern
		Breast cancer metastasis: Often higher uptake reflecting tumor metabolism

FDG, fluorodeoxyglucose; SAPHO, Synovitis, Acne, Pustulosis, Hyperostosis, Osteomyelitis

A PubMed search using the keywords “SAPHO syndrome” and “breast cancer” identified 4 cases where differentiation of lesions from breast cancer bone metastases was problematic. Data for 2 of these cases were freely accessible and are summarized together with the present case (**Table 2**). The full texts of the remaining 2 reports were not accessible, and only limited information could be obtained from the abstracts, precluding their inclusion in **Table 1**. Two patients, including the present case, had skin lesions or a relevant medical history. Imaging revealed that all 3 patients had lesions in the sternoclavicular region. No bone biopsies were performed for definitive diagnosis, but diagnoses were made based on a combination of clinical symptoms and imaging findings.^11,12)^

**Table 2:** Reported SAPHO syndrome cases requiring differential diagnosis with breast cancer metastasis

No.	Reporting year	Reporter	Age	Skin lesions (including past history)	Bone scintigraphy	Bone biopsy
1	2016	Miguel et al.	64	None	Sternal pedicle	None
2	2018	Devin et al.	57	Palmoplantar pustulosis, facial acne	Left sternoclavicular joint, bilateral first costal manubrium joints	None
3	2025	This case	81	Palmoplantar pustulosis	Sternoclavicular joint, sternum to distal end of left clavicle, spine, both knee joints, both ankle joints	None

In SAPHO syndrome, bone biopsies often reveal only nonspecific inflammation, and this low specificity limits the use of biopsy.^13)^ Some reports suggest that bone biopsy is useful for ruling out metastasis when skin lesions are unclear and extra-thoracic bone lesions are prominent.^14)^ However, recent surveys of international clinical practice indicate that bone biopsies are not widely used for the diagnosis of SAPHO syndrome, partly due to the highly invasive nature of the procedure.^13,15)^

In the present case, CT revealed bone sclerosis and hyperplasia in the medial aspects of both first ribs and the sternum. Bone scintigraphy showed abnormal uptake consistent with CT findings of bone enlargement and sclerosis in the sternum, distal left clavicle, spine, and both knee and ankle joints. Bone metastasis from the breast cancer was also suspected. However, the patient had previously experienced pain around the sternoclavicular region and had developed blisters on the palms, diagnosed as palmoplantar pustulosis, which raised the possibility of SAPHO syndrome as a differential diagnosis. The patient had early-stage breast cancer, mucinous carcinoma (cT1bN0M0), which rarely metastasizes to distant sites and has a favorable prognosis.^16,17)^ In addition, previous studies have reported that bone metastases at the time of initial breast cancer diagnosis occur in approximately 3%–4% of patients overall,^18)^ with an incidence of less than 1% per person-year in stage I disease and 1%–3% per person-year in stage II disease,^16)^ indicating that extensive bone metastases at presentation are uncommon in early-stage breast cancer.

Additionally, imaging revealed a bull’s head sign at the sternoclavicular joint, and no other findings suggested metastasis. Therefore, PET-CT and bone biopsy were not deemed helpful for diagnosis and were not performed. After consultation with our hospital’s Radiology, Orthopedic Surgery, and Dermatology departments, the patient was diagnosed with SAPHO syndrome, and treatment for breast cancer was initiated. Nevertheless, the absence of histological confirmation of the bone lesions represents a limitation of this case. Although the clinical history, characteristic imaging findings including the bull’s head sign, and multidisciplinary evaluation strongly supported the diagnosis of SAPHO syndrome, the lack of bone biopsy leaves a degree of diagnostic uncertainty.

Following the diagnosis, the patient was managed according to the standard surveillance protocol for stage IIA breast cancer. During the 16-month follow-up period, no radiological or clinical findings suggestive of metastatic progression were observed. This clinical course is consistent with the initial diagnostic assessment and underscores the importance of appropriate differential diagnosis in similar cases.

Careful differential diagnosis of SAPHO syndrome in patients with breast cancer is paramount, since misdiagnosis of lesions as bone metastases can lead to unnecessary additional chemo- or endocrine therapy.

In breast cancer management, particularly for early-stage breast cancer or subtypes with low metastatic risk, it is crucial not to diagnose bone metastasis based solely on imaging of bone lesions. SAPHO syndrome should be considered in the differential diagnosis when symmetrical sclerosis is observed in the sternoclavicular joints and sternum. Meticulously obtaining a history of skin symptoms and joint pain can help avoid unnecessary treatments.

## CONCLUSIONS

We report a case of breast cancer complicated by SAPHO syndrome, where differentiation of lesions from bone metastases was challenging. SAPHO syndrome should be considered as a differential diagnosis when multiple bone lesions are found in patients with early-stage breast cancer, especially if they present with characteristic imaging findings or skin lesions, such as palmoplantar pustulosis. In evaluating sclerotic bone lesions in patients with breast cancer, a comprehensive assessment of skin symptoms, medical history, and the temporal evolution of imaging findings is crucial for accurate diagnosis and to minimize invasive tests.
